# Nano Ellagic Acid Counteracts Cisplatin-Induced Upregulation in OAT1 and OAT3: A Possible Nephroprotection Mechanism

**DOI:** 10.3390/molecules25133031

**Published:** 2020-07-02

**Authors:** Thikryat Neamatallah, Nagla El-Shitany, Aymn Abbas, Basma G. Eid, Steve Harakeh, Soad Ali, Shaker Mousa

**Affiliations:** 1 Department of Pharmacology and Toxicology, Faculty of Pharmacy, King Abdulaziz University, Jeddah 21589, Saudi Arabia; taneamatallah@kau.edu.sa (T.N.); Beid@kau.edu.sa (B.G.E.); 2 Department of Pharmacology and Toxicology, Faculty of Pharmacy, Tanta University, Tanta 31511, Egypt; 3 Special Infectious Agents Unit, King Fahd Medical Research Center, King Abdulaziz University, Jeddah 21589, Saudi Arabia; aymntalat2001@yahoo.co.ukfg (A.A.); sharakeh@gmail.com (S.H.); 4 Biotechnology Research Laboratories, Gastroenterology Surgery Center, Mansoura University, Mansoura 35511, Egypt; 5 Yousef Abdullatif Jameel Chair of Prophetic Medicine Application, King Abdulaziz University, Jeddah 21589, Saudi Arabia; shaker.mousa@acphs.edu; 6 Anatomy Department of Cytology and Histology, Faculty of Medicine, King Abdulaziz University, Jeddah 21589, Saudi Arabia; soadshaker@gmail.com

**Keywords:** cisplatin, ellagic acid nano, nephrotoxicity, organic anion transporters, oxidative stress

## Abstract

Cisplatin is an anticancer drug commonly used for solid tumors. However, it causes nephrotoxicity. OAT1 and OAT3 are organic anion transporters known to contribute to the uptake of cisplatin into renal tubular cells. The present study was designed to examine the protective role of ellagic acid nanoformulation (ellagic acid nano) on cisplatin-induced nephrotoxicity in rats, and the role of OAT1/OAT3 in this effect. Four groups of male Wistar rats were used (n = 6): (1) control, (2) cisplatin (7.5 mg/kg single dose, intraperitoneal), (3) cisplatin + ellagic acid nano (1 mg/kg), and (4) cisplatin + ellagic acid nano (2 mg/kg). Nephrotoxic rats treated with ellagic acid nano exhibited a significant reduction in elevated serum creatinine, urea, and oxidative stress marker, malondialdehyde (MDA). Additionally, ellagic acid nano restored renal glutathione (GSH), superoxide dismutase (SOD), catalase (CAT), and glutathione peroxidase (GPx). Ellagic acid nano improved the histopathological changes induced by cisplatin, such as tubular dilatation, necrosis, and degeneration. Interestingly, OAT1 and OAT3 showed significantly lower expression at both mRNA and protein levels following ellagic acid nano treatment relative to the cisplatin-exposed group. These findings reveal a potential inhibitory role of ellagic acid antioxidant on OAT1 and OAT3 expression and thus explains its nephroprotective effect against cisplatin nephrotoxicity.

## 1. Introduction

Cisplatin is a chemotherapeutic drug commonly utilized in many cancer chemotherapy treatment regimens [1]. It is highly effective against neuroblastoma as well as various solid tumors, including testicular, breast, ovarian, colorectal, and lung cancers. [1,2]. Cisplatin contains a platinum group which forms covalent adducts with bases in DNA, resulting in the apoptotic death of cancer cells, as well as other rapidly dividing cells [3]. Cisplatin also results in the activation of a cascade of proinflammatory interleukins [4], causes nitrosative and oxidative stress [5,6], and activates p53 protein which induces apoptosis [3,7,8]. Although cisplatin is an effective anticancer agent, it causes liver and kidney toxicities, as well as testicular toxicity and ototoxicity [9,10,11]. Concerning nephrotoxicity, several studies reported dose-dependent acute renal failure in more than 50% of the patients. In addition, about 30% of patients may suffer serious acute kidney injury (AKI) [1,12]. Other documented renal manifestations include Fanconi-like syndrome [13] and thrombotic microangiopathy [14,15].

Cisplatin can penetrate proximal tubular cells through transporters found in the basolateral plasma membrane, mainly the organic anion transporters 1 and 3 (OAT1 and OAT3). Both transporters mediate organic anion/α-ketoglutarate exchange [16,17,18]. Several studies highlighted the importance of OAT1 and OAT3 in such renal conditions as acute renal failure [19] and ureteral obstruction [20]. Interestingly, OAT1/OAT3 knockout mice showed marked protection from renal damage induced by cisplatin [17,21].

Ellagic acid, which is found in nuts and berries, is known to have an antioxidant effect [22]. It exhibits potent reactive oxygen species (ROS) scavenging activity [23,24], as well as other biological effects, such as anticancer [25] and anti-inflammatory [10,26] activities. Recently, the study published by our team showed an immunomodulator protective action of ellagic acid nanoformulation (ellagic acid nano) in hepatic toxicity caused by cisplatin in rats, without compromising the cytotoxic effect of cisplatin [24]. The role of ellagic acid on OAT1 and OAT3 expression in cisplatin-induced nephrotoxicity has not been extensively studied.

Therefore, this study aimed to examine the nephroprotective action of ellagic acid nano on kidney toxicity and oxidative stress. Second, we sought to elucidate the modulatory effect of ellagic acid nano on OAT1 and OAT3 expression.

## 2. Results

### 2.1. Impact of Ellagic Acid Nano on Renal Hypertrophy, Serum Creatinine, and Urea Measured in Nephrotoxic Rats

To demonstrate the effect of ellagic acid nano on renal function after cisplatin exposure, we examined renal hypertrophy, serum creatinine, and urea levels. Intraperitoneal injection of cisplatin (7.5 mg/kg) caused a significant rise in renal hypertrophy, creatinine, and urea levels in the serum relative to controls (*p* ≤ 0.05). However, pretreating the rats with ellagic acid nano (1 mg/kg) significantly reduced hypertrophy, creatinine, and urea levels to 31.5%, 55%, and 41.8%, respectively, relative to the cisplatin group. Ellagic acid nano at the higher dose (2 mg/kg) also decreased the three parameters by 65.1%, 66.3%, and 83.6%, respectively. There was no statistically significant difference between the two ellagic acid nano doses with regard to renal hypertrophy and creatinine, while the high dose excelled in reducing urea (Table 1). These data suggested that the newly formulated ellagic acid nano could prevent cisplatin-induced negative effects on renal hypertrophy, creatinine, and urea.

### 2.2. Impact of Ellagic Acid Nano on Kidney Histopathology Examined in Nephrotoxic Rats

**Figure 1:**
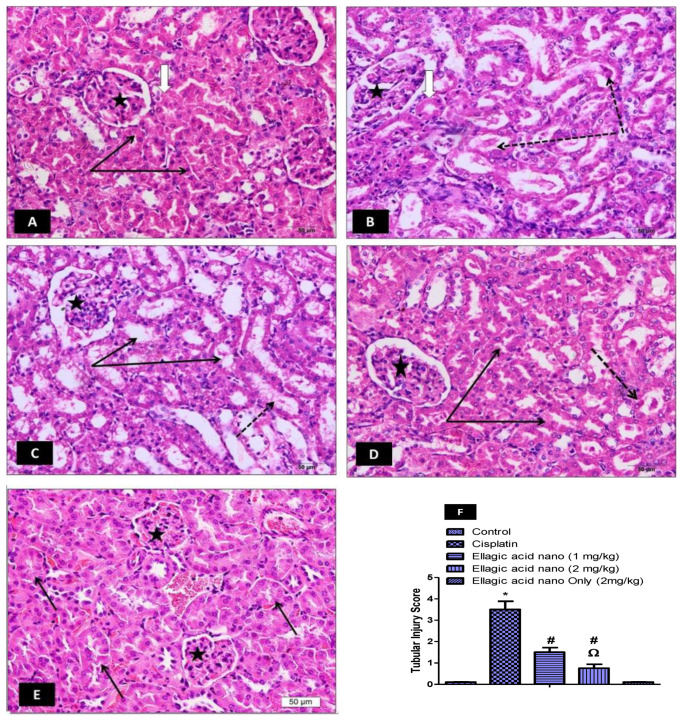
Impact of ellagic acid nanoformulation on kidney histopathology examined in cisplatin- (7.5 mg/kg) treated rats. (**A**) Control: normal kidney parenchyma, renal corpuscle, glomeruli (white arrow and star), narrow lumen proximal tubules, and normal epithelial lining (black arrows). (**B**) Cisplatin: dilated proximal tubules with lumen containing pretentious casts (dotted arrows). Other tubules exhibited dark apoptotic cells with degenerated nuclei (white arrow). Mild changes were observed in the renal glomeruli (stars). (**C**) Ellagic acid nano (1 mg/kg): mildly dilated lumen proximal tubules and renal glomeruli (stars), also few containing casts (dotted arrow). (**D**) Ellagic acid nano (2 mg/kg): marked improvement in proximal tubules (black arrows). This section looked mostly free from casts (dotted arrow). (**E**) Ellagic acid nano only (2 mg/kg): normal renal corpuscles with their glomeruli (stars) and normal renal tubules (black arrows). (**F**) Semiquantitative tubular injury score. Results were presented as mean ± SE (n = 6). * *p* ≤ 0.05 relative to the control group; ^#^ *p* ≤ 0.05 relative to cisplatin group; ^Ω^ *p* ≤ 0.05 relative to ellagic acid nano (1 mg/kg) group.

To further confirm the protective effect of ellagic acid nano on cisplatin-induced renal toxicity, we studied kidney histology of our rat model. In Figure 1A, the control kidney showed normal parenchyma, renal corpuscle, and glomeruli. In addition, proximal tubules exhibited a narrow lumen and healthy epithelial lining. Injecting the rats with cisplatin showed mild changes in the glomeruli and dilated proximal tubules with cast presented in the lumen. Other tubules showed dark apoptotic cells with degenerated nuclei. Furthermore, cisplatin significantly increased the semiquantitative tubular damage score compared to the control group (Figure 1B,F). Pretreatment with ellagic acid nano (1 mg/kg) caused mild changes in the glomeruli, and most proximal tubules displayed dilated lumen; however, they were free from casts (Figure 1C). Kidneys from rats treated with ellagic acid nano (2 mg/kg) manifested with normal renal glomeruli showed marked improvement in most proximal tubules, and were cast-free (Figure 1D). Kidneys from rats treated with ellagic acid nano only showed normal renal glomeruli and normal proximal tubules, which were more active than the control tubule with prominent basal striation, indicating intact membrane infoldings and mitochondria needed for active basal transport (Figure 1E). Ellagic acid nano at both doses significantly improved cisplatin-induced tubular damage, as shown in the semiquantitative tubular damage score (Figure 1F). This experiment further confirmed the protective role of ellagic acid nano with significant favor to the higher dose. In addition, it showed that ellagic acid nano caused no histopathological changes in the kidney, which confirmed its safety.

### 2.3. Impact of Ellagic Acid Nano on Kidney Antioxidants Measured in Nephrotoxic Rats

Oxidative stress plays a key role in cisplatin-induced kidney dysfunction. Since ellagic acid polyphenol exhibits antioxidative properties, we aimed to examine the antioxidant effect (as a protective mechanism) of the new formulation in cisplatin-induced nephrotoxicity. In Table 2, the kidney level of malondialdehyde (MDA), an endproduct of cell membrane lipid oxidative degradation, significantly increased following intraperitoneal (i.p.) injection of cisplatin. In addition, cisplatin decreased the kidney tissue levels of four common antioxidants: glutathione (GSH), glutathione peroxidase (GPx), superoxide dismutase (SOD), and catalase (CAT), relative to the control group (*p* ≤ 0.05). These results confirmed the oxidative stress status induced by cisplatin. Compared to cisplatin, pretreating the rats with ellagic acid nano (1 and 2 mg/kg) significantly reduced kidney MDA, while increasing GSH, GPx, SOD, and CAT levels (*p* ≤ 0.05). Interestingly, there was a significant difference in kidney MDA levels between ellagic acid nano groups, with a significant favor to the higher dose (Table 2). These findings suggest that the protection afforded by ellagic acid nano was attributed at least to its antioxidative properties.

### 2.4. Impact of Ellagic Acid Nano on Kidney Organic Anion Transporter 1 (OAT1) Immunoexpression Examined in Nephrotoxic Rats

**Figure 2:**
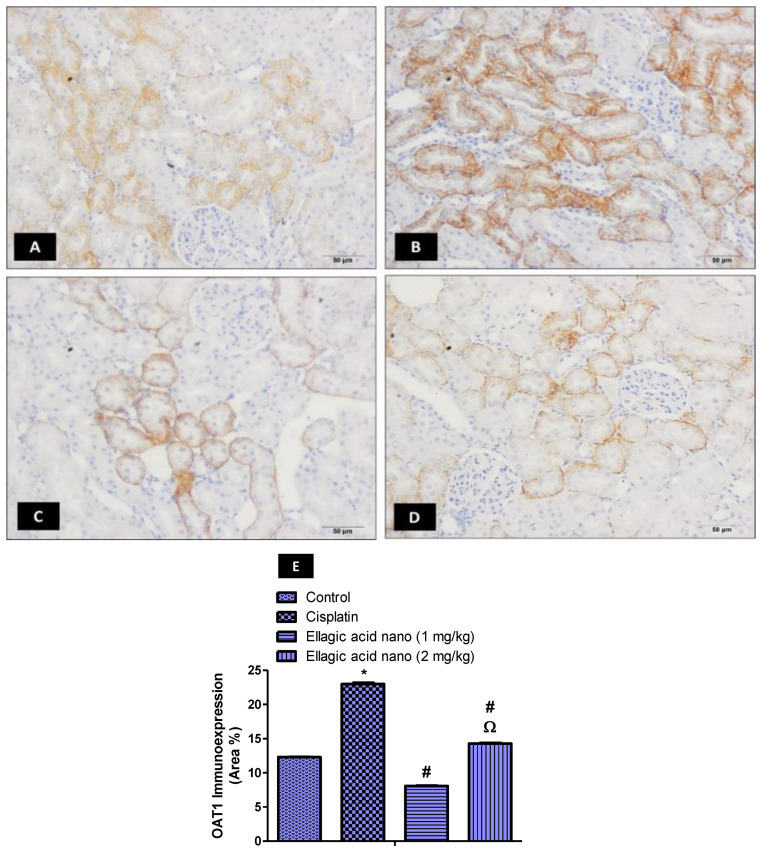
Impact of ellagic acid nanoformulation on kidney OAT1 immunoexpression examined in cisplatin-treated rats. (**A**) Control group; (**B**) cisplatin group; (**C**) ellagic acid nano (1 mg/kg); and (**D**) ellagic acid nano (2 mg/kg). (**E**) Bar chart showing OAT1 immunoexpression (area %) in the different experimental groups. Results were presented as mean ± SE (n = 6). * *p* ≤ 0.05 relative to the control group; ^#^ *p* ≤ 0.05 relative to cisplatin group; ^Ω^ *p* ≤ 0.05 relative to ellagic acid nano (1 mg/kg) group.

The ability of cisplatin to damage kidney tissues is dependent on renal transporters such as OAT1 and OAT3. To demonstrate the contribution of these transporters to ellagic acid nano protection, we examined the expression of OAT1 immunohistochemically in kidney tissues of all rat groups (Figure 2A–D). Intraperitoneal injection of cisplatin at 7.5 mg/kg significantly increased kidney OAT1 immunoexpression, relative to controls (Figure 2B). Interestingly, pretreating the rats with ellagic acid nano (1 and 2 mg/kg) significantly reduced kidney OAT1 expression (65% and 35% reduction, respectively), with a significant favor to the lower ellagic acid nano dose (Figure 2C–E). This suggested that the function of OAT1 can be inhibited by the newly formulated ellagic acid nano and it could be one of the protective mechanisms.

### 2.5. Impact of Ellagic Acid Nano on Kidney Organic Anion Transporter 3 (OAT3) Immunoexpression Examined in Nephrotoxic Rats

**Figure 3:**
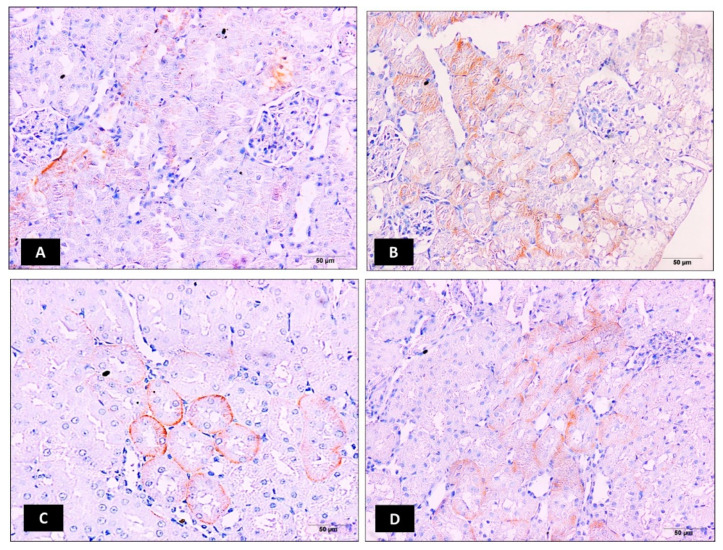

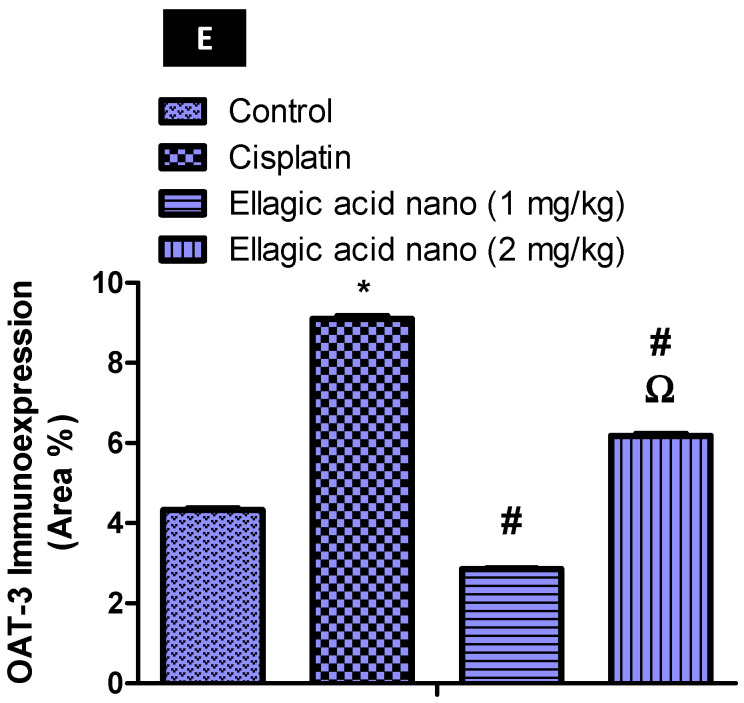
Impact of ellagic acid nanoformulation on kidney OAT3 immunoexpression examined in cisplatin-treated rats. (**A**) Control group; (**B**) cisplatin group; (**C**) ellagic acid nano (1 mg/kg); and (**D**) ellagic acid nano (mg/kg). (**E**) Bar chart showing OAT3 immunoexpression (area %) in the different experimental groups. Results were presented as mean ± SE (n = 6). * *p* ≤ 0.05 relative to the control group; ^#^ *p* ≤ 0.05 relative to cisplatin group; ^Ω^ *p* ≤ 0.05 relative to ellagic acid nano (1 mg/kg) group.

The immunohistochemical expression of OAT3 was also determined and photographed in kidney tissues (Figure 3A–D). Intraperitoneal injection of cisplatin at 7.5 mg/kg significantly increased kidney OAT3 immunoexpression when compared to the control kidney samples *p* ≤ 0.05 (Figure 3B,E). Relative to the cisplatin group, ellagic acid nano (1 and 2 mg/kg) pretreatment significantly reduced kidney OAT3 immunoexpression (65% and 30% reduction, respectively) with a significant favor to the lower ellagic acid nano dose (Figure 3C–E). Similar to OAT1, this suggested that the function of OAT3 can be inhibited by the newly formulated ellagic acid nano and it could be one of the protective mechanisms.

### 2.6. Impact of Ellagic Acid Nano on Kidney Nuclear Factor Kappa-Beta (NFK-B) Immunoexpression Examined in Nephrotoxic Rats

**Figure 4:**
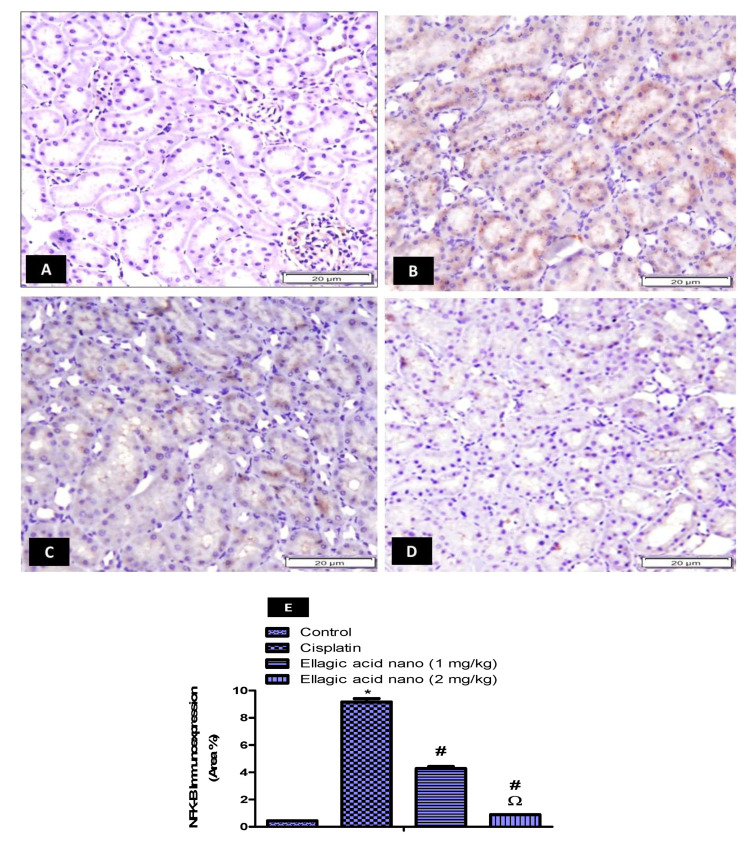
Impact of ellagic acid nanoformulation on kidney nuclear factor kappa-beta (NFK-B) immunoexpression examined in cisplatin-treated rats. (**A**) Control group; (**B)** cisplatin group; (**C**) ellagic acid nano (1 mg/kg); and (**D**) ellagic acid nano (2 mg/kg). (**E**) Bar chart showing NFK-B immunoexpression (area %) in the different experimental groups. Results were presented as mean ± SE (n = 6). * *p* ≤ 0.05 relative to the control group; ^#^ *p* ≤ 0.05 relative to cisplatin group; ^Ω^ *p* ≤ 0.05 relative to ellagic acid nano (1 mg/kg) group.

Cisplatin-induced ROS could stimulate the inflammatory response and cause tissue damage by activating NFK-B. Thus, the immunohistochemical expression of NFK-B was also determined and photographed in kidney tissues (Figure 4A–D). Intraperitoneal injection of cisplatin at 7.5 mg/kg significantly increased kidney NFK-B immunoexpression when compared to the control kidney samples *p* ≤ 0.05 (Figure 4B,E). Relative to the cisplatin group, ellagic acid nano (1 and 2 mg/kg) pretreatment significantly reduced kidney NFK-B immunoexpression (53% and 90% reduction, respectively), favoring the higher dose of ellagic acid nano (Figure 4C–E). Ellagic acid nano at its higher dose significantly decreased MDA (the marker of oxidative damage) and the NFK-B (which was induced by oxidative status) compared to the lower dose. This suggested that ellagic acid nano could ameliorate cisplatin-induced oxidative stress, hence inhibiting NFK-B transcription factor. It was noted that both NFK-B and OATs were expressed in the same site of the kidney, the proximal tubules.

### 2.7. Impact of Ellagic Acid Nano on Kidney mRNA Levels of Organic Anion Transporter 1 (OAT1) Relative Expression to Beta-2-Microglobulin (B2m) Was Examined in Nephrotoxic Rats

**Figure 5:**
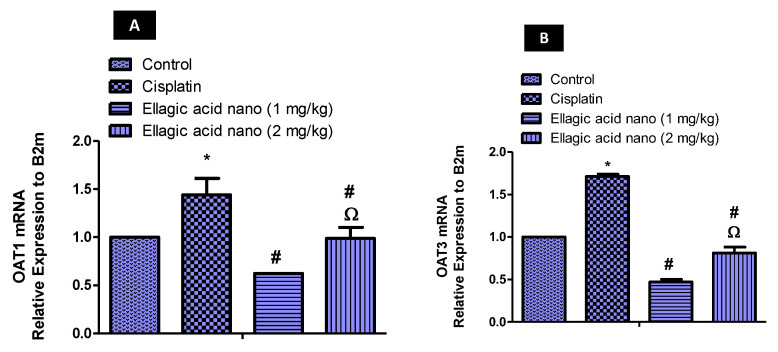
Real-time PCR analysis of (**A**) OAT1 mRNA, and (**B**) OAT3 mRNA expression in kidney tissues of expression levels were normalized to the reference gene B2m using the comparative Ct method (2−∆∆ Ct). Results were presented as mean ± SE (n = 6). * *p* ≤ 0.05 relative to the control group; ^#^ *p* ≤ 0.05 relative to cisplatin group; ^Ω^ *p* ≤ 0.05 relative to ellagic acid nano (1 mg/kg) group.

To further confirm the effect of ellagic acid nano on OATs, the expression of the OAT1 gene was determined using qRT-PCR. Injection of cisplatin (7.5 mg/kg) significantly increased kidney OAT1 mRNA levels compared to the control value (*p* ≤ 0.05). When comparing to the cisplatin group, pretreatment with ellagic acid nano (1 and 2 mg/kg) significantly reduced kidney OAT1 mRNA levels (52% and 31% reduction, respectively). A significant difference was noted in kidney OAT1 mRNA levels favoring the low dose of ellagic acid nano (Figure 5A).

### 2.8. Impact of Ellagic Acid Nanoformulation on Kidney mRNA Levels of Organic Anion Transporter 3 (OAT3) Relative Expression to Beta-2-Microglobulin (B2m) Examined in Nephrotoxic Rats

Similar to OAT1, cisplatin produced a rise in kidney OAT3 mRNA, which was significant in comparison to controls (*p* ≤ 0.05). However, ellagic acid nano (1 and 2 mg/kg) significantly reduced the cisplatin-induced increase in OAT3 mRNA (70% and 55% reductions, respectively). A significant difference was noted in kidney OAT3 mRNA levels favoring the low dose of ellagic acid nano (Figure 5B).

### 2.9. Impact of Ellagic Acid Nano on the Antitumor Activity of Cisplatin

**Figure 6:**
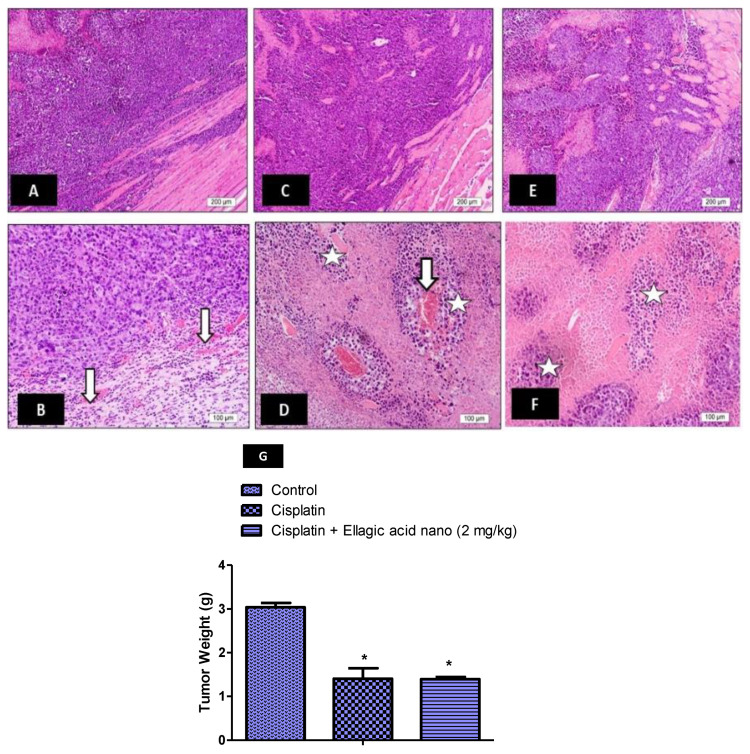
Impact of ellagic acid nanoformulation on solid Ehrlich carcinoma histopathology examined in cisplatin- (3.5 mg/kg) treated rats. (**A**,**B**) Control Ehrlich carcinoma: marked angiogenesis in non-treated solid Ehrlich carcinoma and active malignant cells with marked nuclear pleomorphism (star). (**C**,**D**) Cisplatin: absence of angiogenesis at the tumor periphery (black star) with few blood vessels at the central region (white arrows); notice the degenerated tumor cells with dark pyknotic nuclei (white star). (**E**,**F**) Cisplatin + Ellagic acid nano (2 mg/kg): absence of angiogenesis with marked degenerative changes in tumor cells (white stars). (**G**) A bar chart showing tumor weight in all the experimental groups. Results were presented as mean ± SE (n = 6). * *p* ≤ 0.05 relative to the control Ehrlich carcinoma group.

The effect of ellagic acid nano on the cytotoxic effect of cisplatin was studied using in vivo mice model of Ehrlich solid tumor. Tumor sections of cisplatin-free mice with solid Ehrlich cells showed marked angiogenesis and active malignant cells with marked nuclear pleomorphism (Figure 6A,B). In contrast, tumor sections of cisplatin-treated mice lacked angiogenesis at the tumor periphery, showed few blood vessels at the central region, and degenerated tumor cells with dark pyknotic nuclei (Figure 6C,D). Interestingly, tumor sections of nephrotoxic rats pretreated with ellagic acid nano + cisplatin showed absence of angiogenesis with marked degenerative changes in tumor cells (Figure 6E,F). The sections of mice treated with ellagic acid nano and cisplatin showed more improvement than the those treated with cisplatin alone. Furthermore, the treatment of mice with either cisplatin alone or combined with ellagic acid nano significantly decreased the tumor weight compared to the control Ehrlich group. Combining ellagic acid nano to cisplatin did not influence the tumor weight reduction provoked by cisplatin (Figure 6G). These findings suggested that ellagic acid nano did not affect the anticancer activity of cisplatin in the mice model of Ehrlich solid tumor.

## 3. Discussion

This is the first report to investigate the effect of ellagic acid nano on OAT1/OAT3 expression in cisplatin-induced renal toxicity in rats. Our findings showed that ellagic acid nano counteracted renal injury markers, oxidative stress indicators, and restored renal tissue degeneration induced by cisplatin. The results confirmed that ellagic acid nano improves the antitumor activity of cisplatin in the Ehrlich solid carcinoma in vivo model. This promising effect was accompanied by OAT1 and OAT3 inhibition. Our study results suggest that ellagic acid nano may be combined with other therapies to reduce cisplatin toxicities and patient suffering.

Cisplatin remains an effective anticancer agent. However, it is clinically associated with AKI in 30% of patients [11,27,28]. Cisplatin causes anomalies in kidney structure and function associated with inflammation and excessive generation of ROS [1,8,27]. In the same way, several flavonoids, such as ellagic acid, have shown protection against kidney toxicity induced by cisplatin [16,23,29]. The antioxidant and anti-inflammatory effects previously detected with ellagic acid in vivo and in vitro may play a key role [23,26,29,30]. We recently reported the use of ellagic acid nano, which has superior bioavailability and which may ameliorate cisplatin-induced liver toxicity in rats [24]. We demonstrated the use of ellagic acid nano to overcome nephrotoxicity of cisplatin and used a well-characterized rodent model of kidney injury by injecting a single i.p. dose of cisplatin into rats [23,31,32,33]. Kidney damage was successfully confirmed by histopathological changes along with increased nephrotoxicity markers, creatinine, and urea. In accordance with our findings, previous studies have also shown that cisplatin caused deterioration in the kidney functions via increasing plasma urea and creatinine and decreasing creatinine clearance [32,33]. Ellagic acid nano treatment at both 1 and 2 mg/kg significantly improved renal function and ameliorated histological degeneration of the kidneys by decreasing cisplatin-induced tubular dilation, luminal casts, and glomerular changes.

Cisplatin is activated in the kidney epithelium to highly reactive molecules. Therefore, it induces oxidative stress through the excessive production of ROS, accumulation of MDA, and depletion of antioxidant enzyme activity. GSH, in particular, is an important scavenger of ROS and essential for maintaining cell integrity. SOD, CAT, and GPx are three major antioxidant enzymes that have been found to cause a marked improvement in kidney function. GPx and CAT reduce H_2_O_2_, whereas the dismutation of the superoxide anion (O^2−^) is catalyzed by SOD [34]. Here, oxidative stress induced by cisplatin injection was evidenced by depletion of GSH, GPx, SOD, and CAT levels and a rise in MDA. Interestingly, ellagic acid nano caused an alleviation of oxidative stress and an improvement of the antioxidant status. Similar to our data, the potent scavenging action of ellagic acid on O^2−^ and OH was also reported by Ateşşahín et al. [23], and it represented an important mechanism of protection by ellagic acid. ROS induces oxidative stress and can further result in activation of the NFK-B signaling pathway, which further activates the expression of proinflammatory genes [35,36]. In our work, we showed that cisplatin activated NFK-B as a result of ROS generation and that ellagic acid nano inhibited NFK-B due to amelioration of oxidative stress. This further confirms the antioxidative property of ellagic acid in cisplatin nephrotoxicity.

The ability of cisplatin to enter epithelial cells of the kidney is dependent on OAT1 and OAT3, which mediate the active transport system [16,34]. Both transporters facilitate the uptake of cisplatin mercapturic acid, which is a potent nephrotoxic metabolite [17]. Interestingly, Whitley et al. identified the OAT1 family, human (h)OAT1 and rat (r)OAT1, as an important substrate for ellagic acid [22]. Similar to a previously published study [17], OAT1 and OAT3 mRNA and protein expression were upregulated by cisplatin. This contributes to the uptake of the drug in the tubular cells of the kidney. To our surprise, pretreatment of the nephrotoxic rats with ellagic acid nano produced significant inhibition of OAT1 and OAT3 mRNA and protein expression. Given that both transporters assist cisplatin transport to the kidney cells, any reduction in expression results in decreased uptake of cisplatin. Therefore, strategies to inhibit the expression of OATs should be considered as a defensive mechanism to protect the kidneys against cisplatin injury. Similarly, several other flavonoids may inhibit OAT1 and serve as nephroprotective agents, including fisetin, luteolin, morin, and quercetin [16].

In this study, the anticancer effect of cisplatin was examined in the presence of ellagic acid nano utilizing the mice model of Ehrlich solid carcinoma. The results revealed that ellagic acid nano did not affect the antitumor effect of cisplatin. The current findings are inconsistent with our recently reported findings which confirmed that ellagic acid nano did not influence the cytotoxic activity of cisplatin against the human colorectal cancer cell line (HCT116) [24]. Our data also showed an improvement in the antitumor effect in the mice treated with cisplatin in combination with ellagic acid nano. It was previously reported that ellagic acid inhibited tumor growth and metastasis by suppressing tumor cell proliferation, promoting apoptosis, and preventing angiogenesis [35].

This study showed for the first time that ellagic acid nano offers protection from cisplatin-induced toxicity to the kidney without compromising the cytotoxic effect of cisplatin. This effect may be mediated by the inhibition of organic anion transporters OAT1 and OAT3. These preclinical findings bring ellagic acid to light as a nephroprotective agent against cisplatin and other platinum-based agents.

## 4. Materials and Methods

### 4.1. Chemicals

Cisplatin vials (1 mg/mL) were purchased from Mylan Institutional LLC, Rockford, IL, USA. Ellagic acid nano has been prepared and fully characterized, as reported in our recently published research [24]. Ellagic acid nano was dissolved in sterile distilled water.

### 4.2. Animals

Thirty adult (80–90 days of age) male Sprague Dawley rats (150–180 g) were purchased from King Fahad Research Center, King Abdulaziz University, Saudi Arabia. The rats were maintained for one week to acclimatize at standard laboratory conditions with access to standard food and water. Groups of rats were randomly classified into (n = 6): (1) control: this group was injected with normal saline, (2) cisplatin: rats in this group were injected with cisplatin (7.5 mg/kg, i.p., single dose) [25], (3) ellagic acid nano (1 mg/kg): rats in this group were daily administered ellagic acid nano (1 mg/mL, by oral gavage) for 1 week before cisplatin, (4) ellagic acid nano (2 mg/kg): rats in this group were daily administered ellagic acid nano (2 mg/kg, by oral gavage) for 1 week before cisplatin, (5) ellagic acid nano (2 mg/kg): rats in this group were daily administered ellagic acid nano (2 mg/kg, by oral gavage) for 1 week. The study protocol was performed according to the guidelines of the Ethical Committee, Faculty of Pharmacy, King Abdulaziz University, Saudi Arabia (reference number, PH-123-40).

### 4.3. Samples Collection

Under ether anesthesia, blood was collected by heart puncture technique at the end of the experiment (72 h post-cisplatin) and centrifuged to separate the serum for 10 min at 1008 g.

The two kidneys were removed from the anesthetized rats. The thin capsule was stripped out. The right kidney was chosen for histopathology (as it has venous drainage direct to posterior vena cava), and opened along its convex surface into two halves. One half was immersed into 10% neutral buffered formalin for 24 h before paraffin processing. The other was immediately excised and kept at (−80 °C) until analyzed.

Renal hypertrophy was calculated according to [37] by the following formula:

$$\mathrm{Renal}\;\mathrm{hypertrophy}\;=\frac{\mathrm{Kidney}\;\mathrm{weight}\;\left(g\right)}{\mathrm{Body}\;\mathrm{weight}\;\left(g\right)}\times 1000$$

### 4.4. Measurements of Serum Creatinine and Urea Levels

Serum creatinine and urea levels were determined in the experimental groups using Crescent Diagnostics kits (Crescent Diagnostics, Saudi Arabia) according to the manufacturer’s instructions.

### 4.5. Examination of Kidney Histopathology

The formalin preserved kidneys were sliced into 5 μm sections, fixed on a glass slide, and further stained with hematoxylin and eosin (H & E). A light microscope (Nikon Eclipse TE2000-U, Nikon, Japan) was used to examine and photograph the slides.

Renal tubular damage (tubular dilation, apoptosis, necrosis, and cast) in H & E sections was scored using a previously described semiquantitative scale [38]. The pathologist was absolutely a blinded reviewer.

### 4.6. Measurements of Kidney Oxidative Stress/Antioxidants Markers

The kidney was weighed and homogenized immediately to obtain 50% (w/v) homogenate in ice-cold phosphate buffer (pH 7.4) + 2% Triton X-100, then centrifuged at 500× *g* for 10 min at 4 °C. The supernatant was stored at −80 °C for the measurement of oxidative stress measures and antioxidant enzymes [39].

Malondialdehyde (MDA), reduced glutathione (GSH), glutathione peroxidase (GPx), superoxide dismutase (SOD), and catalase (CAT) activity were determined in the kidney homogenates using Biodiagnostic kits, Egypt.

### 4.7. Immunohistochemistry Localization and Quantification of OAT1, OAT3, and NFK-B Gene in the Kidney

An immunoperoxidase (peroxidase/antiperoxidase, PAP) protocol was employed to stain the kidney sections. OAT1 (ab131087, Abcam, Cambridge, UK), OAT3 (sc-293264, Santa Cruz Biotechnology, Inc., Dallas, TX, USA), and NFK-B (RB-9034-R7, Lab Vision, Fremont, CA, USA) antibodies were used at a dilution of 1:200. A light microscope was used to examine and photograph the slides, and quantification was performed with ImageJ software (ImageJ, 1.46a, NIH, USA).

### 4.8. Quantitative Real-Time Polymerase Chain Reaction (qRT-PCR) for Determination of mRNA Expression of OAT1 and OAT3 Genes

The manufacturer’s protocol was followed for total RNA extraction from the kidney tissues by RNA extraction (Applied Biosystems, Foster City, USA). A cDNA synthesis kit (Applied Biosystems, USA) was used for RNA transcription. Table 3 shows the primer nucleotide sequences used. The qRT-PCR assay was carried out using SYBR Select Master Mix (Applied Biosystems, USA). Firstly, uracil-DNA glycosylase activation of 2 min at 50 °C was carried out. This was followed by the activation of DNA polymerase for 2 min at 95 °C. Next, 40 cycles of 15 s at 95 °C, 60 s at 60 °C were carried out. Finally, 5 min at 72 °C followed for extension, and the reaction was stopped by incubation and cooling to 4 °C. After amplification, a melt-curve analysis was performed. The relative quantification (∆∆Ct) method was adopted to validate the results [40]. The average of three runs for each gene was normalized in relation to the average of beta-2 microglobulin (B2m).

### 4.9. Impact of Ellagic Acid Nano on the Antitumor Activity of Cisplatin

The impact of ellagic acid nano on the antitumor activity of cisplatin was evaluated in vivo in mice. A xenograft model of solid Ehrlich carcinoma was induced in all mice by implanting 2 × 10^6^ viable Ehrlich ascites carcinoma cell line cells suspended in 0.2 mL isotonic saline. Ehrlich ascites carcinoma cells were implanted subcutaneously in the thigh of each mouse. The tumor developed in 100% of mice with a palpable solid tumor mass achieved within 10 days post-implantation [41]. Three groups of female Swiss albino mice (n = 6) bearing solid Ehrlich cells were utilized in this experiment. At day zero after the inoculation, the mice were subjected to the following treatment protocol: (1) Ehrlich control group (mice treated with normal saline i.p. daily), (2) cisplatin group (mice treated with a single dose of cisplatin 3.5 mg/kg, i.p.) [42], and (3) ellagic acid nano + cisplatin group (mice treated with ellagic acid nano 2 mg/kg, by oral gavage daily). The treatment protocol for all groups was started on day zero and extended to day 14 post-implantation. On day 14 post-implantation, all surviving mice were sacrificed. The tumor was excised, washed immediately with ice-cold saline, weighed, and the specimen was preserved in 10% formalin solution. The samples were stained with H & E and examined for the tumor state.

### 4.10. Statistical Analysis

ANOVA followed by the Tukey HSD post-hoc test were used for statistical comparison, using GraphPad Prism version 5. The results were given as mean ± standard error (SE); *p* ≤ 0.05 was regarded as significant.

## Figures and Tables

**Table 1:** Impact of ellagic acid nanoformulation on renal hypertrophy, serum creatinine, and urea measured in cisplatin-treated rats.

	Control	Cisplatin	Ellagic Acid Nano (1 mg/kg)	Ellagic Acid Nano (2 mg/kg)
Renal hypertrophy	4.15 ± 0.05	5.96 ± 0.26 ^a^	4.08 ± 0.41 ^b^	3.88 ± 0.55 ^b^
Creatinine (mg/dL)	0.65 ± 0.05	2.38 ± 0.18 ^a^	1.07 ± 0.17 ^b^	0.80 ± 0.07 ^b^
Urea (mg/dL)	26.9 ± 1.41	181.20 ± 8.09 ^a^	105.32 ± 6.01 ^b^	29.53 ± 3.69 ^b, c^

Results were presented as mean ± SE (n = 6). ^a^ *p* ≤ 0.05 relative to the control group. ^b^ *p* ≤ 0.05 relative to the cisplatin group. ^c^ *p* ≤ 0.05 relative to ellagic acid nano (1 mg/kg) group.

**Table 2:** Impact of ellagic acid nanoformulation on kidney antioxidants measured in cisplatin-treated rats.

	Control	Cisplatin	Ellagic Acid Nano (1 mg/kg)	Ellagic Acid Nano (2 mg/kg)
MDA (μM/mg protein)	35.5 ± 9.7	113.5 ± 12.1 ^a^	62.8 ± 3.5 ^b^	25.9 ± 6.9 ^b, c^
GSH (mg/mg protein)	4.6 ± 0.17	3.9 ± 0.18 ^a^	5.1 ± 0.34 ^b^	5.4 ± 0.24 ^b^
GPx (U/mg protein)	665 ± 39	136 ± 11 ^a^	221 ± 7 ^b^	241 ± 4 ^b^
SOD (U/mg protein)	1615 ± 270	373 ± 72 ^a^	600 ± 53 ^b^	605 ± 41 ^b^
CAT (U/mg protein)	13.0 ± 2.1	6.7 ± 0.5 ^a^	9.6 ± 0.9 ^b^	10.5 ± 0.4 ^b^

Results were presented as mean ± SE (n = 6). ^a^ *p* ≤ 0.05 relative to the control group. ^b^ *p* ≤ 0.05 relative to the cisplatin group. ^c^ *p* ≤ 0.05 relative to ellagic acid nano (1 mg/kg) group.

**Table 3:** Primer nucleotide sequences used in the qRT-PCR examination of gene expression.

Primer Name	Sequence
B2m	Forward: 5′- GATGTCAGATCTGTCCTTCAGCA -3′ Reverse: 5′- GTCTCGGTCCCAGGTGACG -3′
OAT1	Forward: 5′- CGTCGGACGCTTCCAGTTGA -3′ Reverse: 5′- CTCCAGACCTCCATCTTTGCT -3′
OAT3	Forward: 5′- TGCCTACTACAGTTTGGCTATGG -3′ Reverse: 5′- AGGAGCAGGAGGAAGCTCTG -3′
