# Correction: Neamatallah et al. Nano Ellagic Acid Counteracts Cisplatin-Induced Upregulation in OAT1 and OAT3: A Possible Nephroprotection Mechanism. *Molecules* 2020, *25*, 3031

**DOI:** 10.3390/molecules28155626

**Published:** 2023-07-25

**Authors:** Thikryat Neamatallah, Nagla El-Shitany, Aymn Abbas, Basma G. Eid, Steve Harakeh, Soad Ali, Shaker Mousa

**Affiliations:** 1 Department of Pharmacology and Toxicology, Faculty of Pharmacy, King Abdulaziz University, Jeddah 21589, Saudi Arabia; 2 Department of Pharmacology and Toxicology, Faculty of Pharmacy, Tanta University, Tanta 31511, Egypt; 3 Special Infectious Agents Unit, King Fahd Medical Research Center, King Abdulaziz University, Jeddah 21589, Saudi Arabia; 4 Biotechnology Research Laboratories, Gastroenterology Surgery Center, Mansoura University, Mansoura 35511, Egypt; 5 Yousef Abdullatif Jameel Chair of Prophetic Medicine Application, King Abdulaziz University, Jeddah 21589, Saudi Arabia; 6 Anatomy Department of Cytology and Histology, Faculty of Medicine, King Abdulaziz University, Jeddah 21589, Saudi Arabia

## Error in Figure

In the original publication, there was a mistake in Figure 3 as published [1]. We observed that photo B was mistakenly presented in the cisplatin group. However, this photo was from the ellagic acid nano (2 mg/kg) group. So, we changed photo B with another photo from the cisplatin group. This doesn’t change the conclusion made. The corrected Figure 3 appears below.

The authors state that the scientific conclusions are unaffected. This correction was approved by the Academic Editor. The original publication has also been updated.

## Figures and Tables

**Figure 3:**
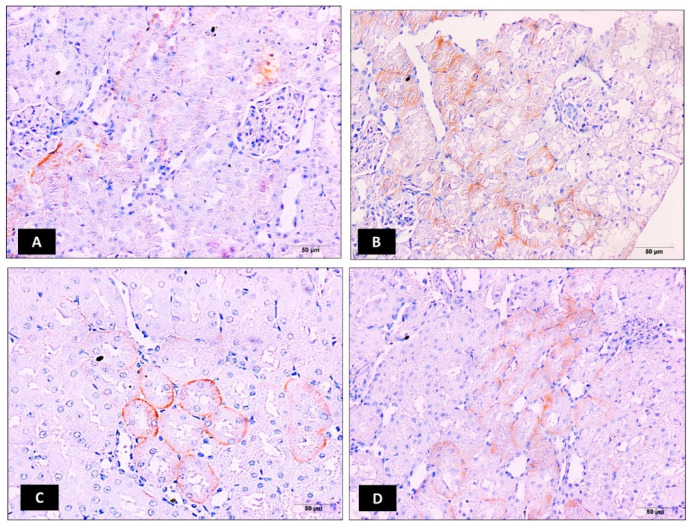

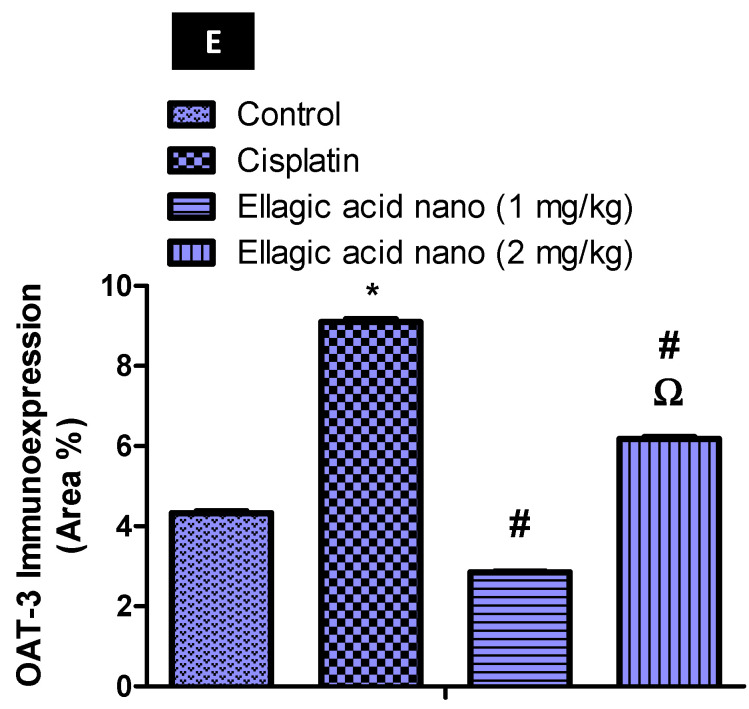
Impact of ellagic acid nanoformulation on kidney OAT3 immunoexpression examined in cisplatin-treated rats. (**A**): Control group; (**B**): Cisplatin group; (**C**): Ellagic acid nano (1 mg/kg); and (**D**): Ellagic acid nano (2 mg/kg). (**E**): Bar chart showing OAT3 immunoexpression (area %) in the different experimental groups. Results are given as mean ± SE (n = 6). * *p* ≤ 0.05 relative to the control group; ^#^ *p* ≤ 0.05 relative to cisplatin group; ^Ω^ *p* ≤ 0.05 relative to ellagic acid nano (1 mg/kg) group.
